# Beyond propulsion: muscle proprioception enables hydrodynamic sensing in fish body

**DOI:** 10.1098/rspb.2025.0474

**Published:** 2025-10-29

**Authors:** Rahdar Hussain Afridi, Waqar Hussain Afridi, Muhammad Hamza, Mingxin Wu, Li-Ming Chao, Yufan Zhai, Liang Li, Guangming Xie

**Affiliations:** ^1^ State Key Laboratory for Turbulence and Complex Systems, Intelligent Biomimetic Design Laboratory, College of Engineering, Peking University, Beijing, People’s Republic of China; ^2^ School of Mechanics and Safety Engineering, Zhengzhou University, Zhengzhou, Henan, People’s Republic of China; ^3^ Department of Mechanics, Inner Mongolia University of Technology, Hohhot, Inner Mongolia; ^4^ Department of Collective Behaviour, Max Planck Institute of Animal Behavior, Konstanz, Germany; ^5^ Centre for the Advanced Study of Collective Behaviour, University of Konstanz, Konstanz, Germany; ^6^ Department of Biology, University of Konstanz, Konstanz, Germany; ^7^ Department of Computer and Information Science, University of Konstanz, Konstanz, Germany; ^8^ Institute of Ocean Research, Peking University, Beijing, People’s Republic of China

**Keywords:** proprioceptive sensing, hydrodynamics in fish swimming, EMG to kinematics mapping

## Abstract

In aquatic environments, muscle activity in free-swimming fishes not only propels body undulations to generate thrust but also serves as proprioceptive sensors for detecting surrounding fluid dynamics. Testing the proprioceptive function of the muscle is challenging owing to its deep integration with swimming activity. To address this, we introduce an experimental platform that records up to 12-channel electromyography (EMG) signals synchronized with detailed kinematics in koi and carp. We first apply various neural networks to map densely collected EMG signals to synchronized video-based body kinematics, thereby validating our EMG collection system. We then compare EMG data from fishes swimming in various laminar flows and within Kármán vortices. Our results show that the phase of muscle activity consistently precedes body kinematics in various laminar flows. While within Kármán vortices, we observe a mixed phase relationship, where muscle activity sometimes leads and at other times lags behind body kinematics. This suggests that fishes may use muscle proprioceptive sensing when interacting with complex flows, such as nearby vortices. Our research not only introduces novel methods for biological EMG studies but also offers insights that could influence the design of bio-inspired underwater sensory systems.

## 1. Introduction

Fishes have evolved an efficient and agile sensory-motor system for swimming, incorporating vision, lateral line sensing, olfaction and auditory functions [1–6]. Increasing evidence suggests that fishes may use proprioceptive sensing to detect nearby hydrodynamics and adapt their body movements accordingly [7–10]. For instance, neurons in a dead fish’s fin will fire if the fin is bent, such as by nearby flow [11]. Proprioceptive sensing alone could be sufficient for fishes to ‘sense’ nearby flow and adjust their body phase to harness energy from the flow [5,6,8]. However, owing to the strong coupling between proprioceptive sensing and active body movements [9,12–14], testing this hypothesis in living systems—while they interact with the flow to determine whether fishes truly gain and apply proprioceptive sensing in adjusting their gait—remains challenging.

Previous studies have applied neuron recording to study proprioceptive sensing in fishes [10,15]. For example, using intracellular and extracellular electrodes to record neural activity from proprioceptive receptors and their innervating nerves during controlled fin bending, Williams *et al*. demonstrated that fishes can detect proprioceptive signals in response to fin deformation [10]. Using electrophysiological recordings from the lateral line system, Skandalis *et al*. [16] found that corollary discharge sharpens sensory feedback during body movements, preventing sensory adaptation and enhancing proprioceptive accuracy. However, these methods often require the fish to be anaesthetized or immobilized to measure neuronal responses to proprioceptive stimuli under artificial fin bending. Genetic labelling and manipulation is another powerful method for investigating the function of proprioceptive sensing. Piton *et al*. [15] used genetic techniques to manipulate Piezo2 channels in zebrafish and observed significant changes in swimming patterns with and without the knockout of these channels, leading to the identification of a proprioceptive organ in the spinal cord of adult zebrafish. However, these methods are often very complex and have potential off-target effects that might also affect swimming ability. Recently, numerical or physical models have been applied to generate/test proprioceptive sensing hypotheses [10,15,16]. For instance, Li *et al*. used a robotic fish equipped with proprioceptive sensing as the sole feedback mechanism and found it sufficient for the fish to extract energy from the vortices shed by neighbouring fishes—a phenomenon known as vortex phase matching, which does not necessarily require vision or lateral line sensing [8]. While these models can offer valuable hypotheses and even potential mechanisms for proprioceptive sensing in swimming fishes, they are extremely difficult to test in biological systems. Therefore, we still need a straightforward yet powerful method to test proprioceptive sensing of hydrodynamics in freely swimming fishes.

Electromyography (EMG) presents a promising solution, as it captures muscle activity, which reflects proprioceptive signals [12,13,16,17], and directly measures key details such as the timing, frequency and intensity of muscle movements [18]. Previous studies have primarily used EMG to examine the roles of red and white muscles in swimming [5,19–21] and to assess the energy costs of fishes swimming under various conditions [5]. For instance, by comparing EMG signals from fishes swimming in still water and within Kármán vortices, researchers found that fishes use less energy when swimming in the vortices [22]. While EMG has been valuable for understanding muscle activity in animals, it has relatively fewer channels, generally up to eight channels or fewer [23–26], which, while sufficient for basic muscle activity analysis, may lack the resolution needed for in-depth proprioceptive studies. Additionally, the collected EMG signals are unsynchronized with body kinematics, thus limiting the insights into the activation and perception function of the muscle in fish swimming.

In this article, we investigated proprioceptive sensing in fish swimming by recording EMG signals from fish muscles under various flow conditions. Specifically, since fish swimming is primarily driven by muscle activity, observable body kinematics should typically lag behind the EMG signals [18,19,21,23,27,28]. However, if fishes possess proprioceptive sensing as hypothesized in these physical systems, the sequence may start with kinematic movement, followed by proprioceptor activation and then EMG signals. This pattern should diverge from the typical sequence, where EMG activity precedes kinematics [18,19,21,23,27–34]. To explore this, we developed a custom platform capable of simultaneously recording up to 12-channel EMG signals synchronized with body kinematics captured by a top-view camera. We validated the platform’s performance by applying deep neural network models, including a backpropagation neural network (BPNN), a long short-term memory (LSTM) network, and a convolutional neural network (CNN) to reconstruct detailed kinematics from the EMG data. We finally tested the proprioceptive sensing hypothesis by conducting experiments with six fishes swimming in laminar flow, both with and without cylinders of various sizes and analysing the phase relationship between EMG and body kinematics to test the proprioceptive sensing hypothesis. We found that muscle activities consistently precede body undulations in laminar flow without a cylinder. However, when swimming in Kármán vortices behind a cylinder, the EMG sometimes lags behind the body undulations, indicating that the fish’s body may first bend owing to the nearby flow [3] and the muscles react later, after sensing the body bending proprioceptively. All of this suggests that fishes can adapt their gaits based on proprioceptive sensing through muscle in complex flow environments, such as within vortices.

## 2. Results

### (a) The electromyography collection platform

**Figure 1.**
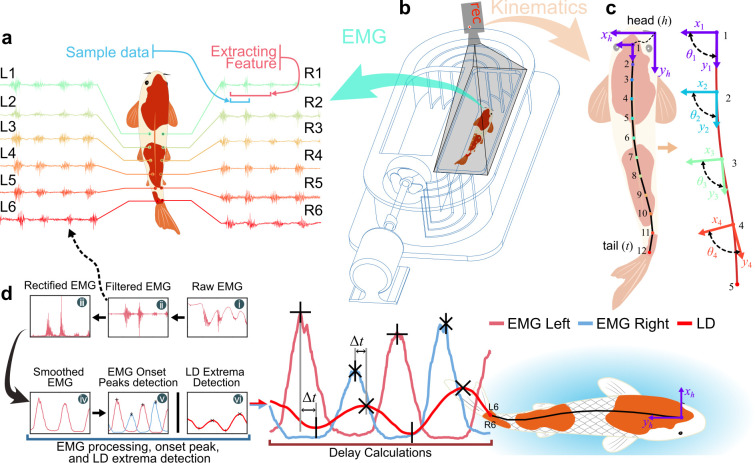
Schematics of the experiment set-up and data processing. (a–c) The fish (a) EMG signals with 12 channels and video are simultaneously recorded in the (b) flow tank for swimming (c) kinematics. (d) EMG signal processing and delay (∆t) calculation pipeline. (i) Raw EMG signals were (ii) filtered, (iii) rectified and (iv) smoothed to extract muscle activation. (v) EMG onset peaks and (vi) lateral displacement (LD) extrema were detected and delays were calculated as the time difference between EMG peaks and nearby LD events.

The proposed platform consists of two main components: EMG collection and synchronized body kinematics recording (figure 1a–c). The EMG set-up uses two bio-signal amplifiers to enhance the small voltages generated by fish muscles; amplifiers process 12 EMG channels from both the left and right sides of the fish (figure 1a; electronic supplementary material, figures S2 and S3). The amplifiers use a differential biosignal amplifier (ED0039), with each channel having specific input electrodes (+, − and reference). Thin-insulated copper wires (0.1 mm diameter and 1.5 m long) are used as electrodes to collect EMG signals from the fish’s body. The ends of these wires are stripped of insulation (10 mm) and bent into a hook shape to ensure they stay inside the fish muscle. A DC power (5 V battery) is applied for the set-up to minimize noise and interference. EMG signals for each channel are acquired using a National Instruments (USB-6210) at a sampling rate of 2.5 k samples s^−1^. Videos were captured using a Sony RX100m5 camera positioned above the flow tank (Loligo®) at 100 fps and a resolution of 1920 × 1080. Detailed body kinematics are extracted from the top-view video (figure 1b) using DeepLabCut (DLC; v2.3) [35] (figure 1c). The EMG signal and kinematic data (video) were simultaneously recorded (figure 1a,c; electronic supplementary material, video M1). We synchronize EMG data collection with body kinematics using an LED indicator that turns on when EMG recording begins and turns off when it ends. This LED signal is captured by the top-view camera recording the body kinematics, ensuring precise alignment between the two datasets (a maximum synchronization error of ±20 ms, see the electronic supplementary material, note S3).

### (b) Validation of electromyography-kinematics collection for testing proprioceptive sensing

**Figure 2.**
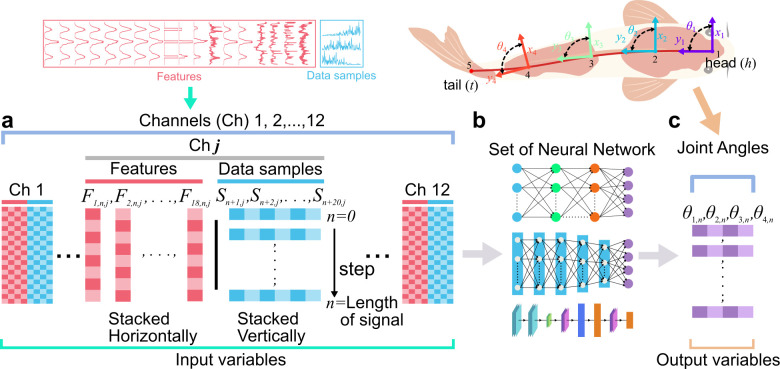
Input–output mapping for set of ML models. (a–c) Eighteen features were extracted from each EMG channel and combined with filtered EMG data samples, (a) pooled as inputs for (b) three different neural networks to predict the (c) four joint angles as the kinematic outputs (see the electronic supplementary material, notes S5–S8).

**Figure 3.**
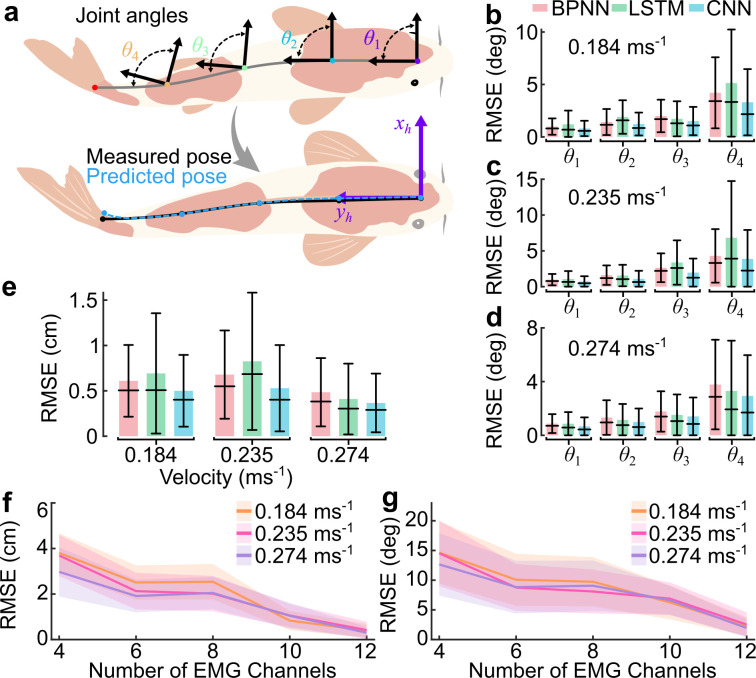
EMG mapping to body kinematics. (a) The illustration of the angles along the body. The predicted angles are transformed into body pose. Then, the predicted body pose’s RMSE is calculated against the measured body pose extracted by DeepLabCut. (b–d) The mean RMSE, along with s.d. and median of each individual predicted angle ($\theta_{1},\theta_{2},\theta_{3},\theta_{4}$) of three ML models for swimming speed of (b) 0.184, (c) 0.235 and (d) 0.274 m s^−1^ (see the electronic supplementary material, equation S9 in note S11 for RMSE calculation). (e) The mean RMSE along with s.d. and median in the predicted fish body pose ($P_{x,pred}$) relative to measured body pose ($P_{x,meas}$) at 0.184, 0.235 and 0.274 m s^−1^ swimming speeds. Only the *x*-component of the body pose is shown, as the error in the *y*-component was found to be negligible owing to near-planar body motion (see the electronic supplementary material, note S11, equation S7, table S4). (f,g) The RMSE of (f) body pose and (g) all predicted angles ($\theta_{1},\theta_{2},\theta_{3},\theta_{4}$) shows the effect of the number of EMG channels on CNN predictions for 4, 6, 8, 10 and 12 channels at swimming speeds of 0.184, 0.235 and 0.274 m s^−1^ (mean RMSE—solid line and s.d. of RMSE—shading; see the electronic supplementary material, equations S10 and S11 in note S11 for RMSE calculation).

To test the proprioceptive sensing hypothesis, it was first essential to validate our platform could reliably capture the spatiotemporal structure of muscle activity. We therefore used machine learning (ML) models to map multi-channel EMG signals to synchronized body kinematics, ensuring that the EMG data reflected meaningful motor output suitable for hypothesis testing. We conducted experiments with a total of 15 fish (koi, *Cyprinus rubrofuscus*, *n* = 10, body length (BL) = 28.15 ± 3.9 cm and carp, *Cyprinus carpio*, *n* = 5, BL = 33.6 ± 2.1 cm), as listed in the electronic supplementary material, table S2. We then verified the EMG/kinematic collection platform with the potential mapping from EMG signals to body kinematics (such as joint angles and positions) with three ML models using data of fish (*n* = 9) swimming in laminar flow (0.1458, 0.1838, 0.2348, 0.2652 and 0.274 m s^−1^). Specifically, we choose BPNN for nonlinear mapping [36], LSTM for handling time dependencies [37], and CNNs for spatial feature extraction [38] (figure 2b; material and methods, electronic supplementary material, figures S5 and S6, and notes S5−S8). We pooled EMG features from the time domain, frequency domain and time-frequency domain, along with rectified EMG data samples as input variables to map the corresponding kinematics, ensuring a comprehensive representation of muscle activity and facilitating temporal and spatial learning in models (see the electronic supplementary material, note S4). We chose four typical joints ($\theta_{1}$, $\theta_{2}$, $\theta_{3}$ and $\theta_{4}$) as inputs variable enough to estimate the curvature of the fish (figure 3a). Each model’s structure is tailored to its purpose: BPNN uses multiple dense layers with up to 512 neurones (electronic supplementary material, figure S5), LSTM employs stacked layers with 64 to 16 units (electronic supplementary material, figure S6), and CNN uses convolutional layers with 32 and 64 filters followed by dense layers (electronic supplementary material, figure S7 and notes S6−S8 for details). To assess the mapping quality, we compare the root mean square error (RMSE) and temporal alignment between the predicted and measured joint angles (figure 3b–d; electronic supplementary material, table S4). Additionally, we reconstruct the full body pose as well as key locomotion metrics from the mapped joint angles (figure 3e; electronic supplementary material, figure S13c–e, table S4). Finally, we investigated how the number and placement of EMG channels affect mapping quality by analysing the RMSE of joint angles and body pose (figure 3f,g; electronic supplementary material, figure S8, table S5, notes S9 and S11 for RMSE calculation).

We first analysed all three models for predicting four joint angles with 12 EMG channels of information (figure 3b–d; electronic supplementary material, figure S9b,d,f). The RMSE between the predicted and measured joint angle for each joint (figure 3b–d; see the electronic supplementary material, equation S9 in note S11 for RMSE calculation). We see the prediction error increases for joints closer to the tail for all three models and at all three different flow speeds (figure 3b with a flow speed of 0.164 m s^−1^, figure 3c with a flow speed of 0.235 m s^−1^, and figure 3d with a flow speed of 0.274 m s^−1^). The s.d. of the error of the predictions is also consistent with this (electronic supplementary material, table S4). This could be owing to the large amplitude near the tail tip. We also found that, overall, out of all three models, CNN gives minimum deviations (mean error 3.87 deg, which is 9% of the tail maximum angle). We then applied forward kinematics to reconstruct the fish’s body pose from predicted joint angles (figure 1a; also see §4), including the lateral body movements, tail-beat frequency (*f*), time period (*T*), amplitude (*A*) and Strouhal number (St) of kinematics based on reconstructed body geometry information (figure 3e; electronic supplementary material, figures S12 and S13 and tables S6 and S7, note S12), such as the BL. We also found that all three models performed well, with the maximum RMSE between predicted and measured body pose being less than 1.88 cm, which corresponds to less than 5.5% BL, and CNN gives the best performance with a maximum RMSE smaller than 1.2 cm (3.5% BL; see the electronic supplementary material, table S4, note S12, equation S7 and S8 in note S11 for RMSE calculation). We further analysed the temporal alignment of the predicted angles and measured joint angles by measuring the phase difference (∆∅; see the electronic supplementary material, note S10). The instantaneous ∆∅(t) is measured as $\Delta \emptyset_{pred}\left(t\right)\;-\;\Delta \emptyset_{meas}\left(t\right)$ with negative values indicating that the predicted angles lags behind the measured angles and positive values indicating it leads (electronic supplementary material, figure S9c,e,g). All models resulted in a $\Delta \emptyset_{degree}$, fluctuating around zero, with a mean ∅ ranging from −0.59° to 2.07° across all conditions, indicating predicted angles and measured angles are temporally aligned (electronic supplementary material, table S4).

Finally, we evaluated how the number of channels affects the mapping from EMG signals to body kinematics. We tested channel numbers ranging from 4 to 12 in intervals of 2 across three different flow speeds. Our results indicate that as the number of channels increases, the RMSE for both position and joint angle decreases (figure 3f,g; electronic supplementary material, figure S8, table S5, note S9), reflecting improved kinematic predictions from the EMG signals. Additionally, the decreasing s.d. of the RMSE values reflects the stability of the predictions, supporting the platform’s robustness for precise kinematic analysis. However, the improvement in pose prediction between 10 and 12 channels is minimal (0.61 ± 0.19 cm), compared with the significant improvement observed between 4 and 10 channels, where the RMSE decreased to 2.51 ± 0.45 cm (electronic supplementary material, table S5). This suggests that it needs a relative number of channels to capture body dynamics effectively. All these EMG-based kinematic property prediction results suggest that our platform is effective, with EMG signals showing a strong correlation to body kinematics (electronic supplementary material, tables S4–S7).

### (c) Test proprioceptive sensing in fish swimming

**Figure 4.**
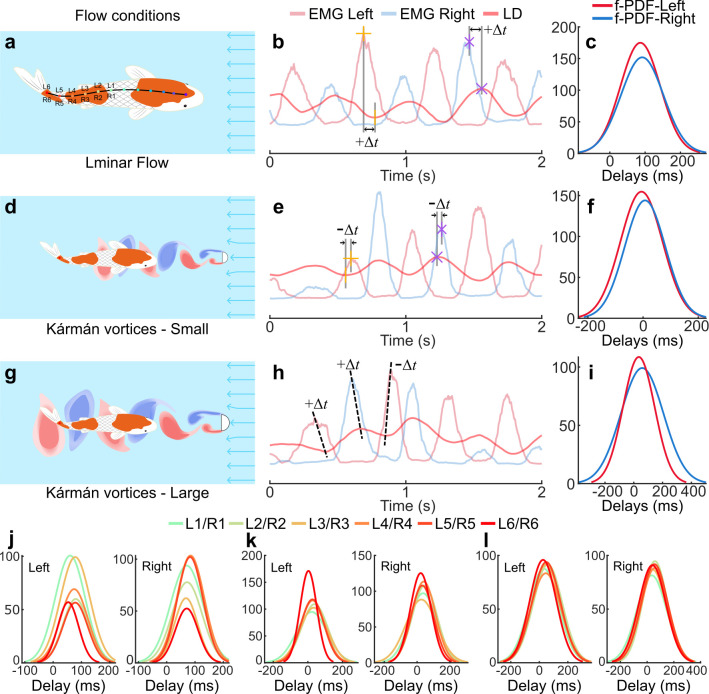
Temporal analysis of EMG and kinematics for proprioceptive sensing in swimming fishes. (a,d,g) The fish EMG is collected while swimming at three flow conditions: (a) laminar flow condition, (d) Kármán vortices (5 cm D-section or cylinder) and (g) Kármán vortices (7 cm D-section). (a) The electrodes placement of channels L1–L6 on the left side and R1–R6 on the right side are positioned along the body at 0.5, 0.6, 0.7, 0.8, 0.9 and 1, respectively. (b,e,h) The EMG of the left (l) and right (r) are time series plotted against the midline point’s lateral displacment (LD) regardless of *y*-axis magnitude to illustrate the calculation of delays ∆t (electronic supplementary material, note S13). (c,f,i) The probability density function (pdf) of the F-distribution (f-PDF) of all delays (L6/R6 versus tail LD) was calculated for several subjects in swimming at different flow conditions: (c) laminar (*n* = 9), (f) Kármán vortices—5 cm (*n* = 6), and (i) Kármán vortices—7 cm (*n* = 6). (j–l) The f-PDF of delays EMG channels (L1−6/R1−6) against respective midline points LD for several subjects swimming in different flow conditions: (j) Laminar condition (*n* = 4), (k) Kármán vortices—5 cm (*n* = 4), and (l) Kármán vortices—7 cm (*n* = 4).

To test the proprioceptive sensing hypothesis, we conducted experiments with 15 fishes (koi, *C. rubrofuscus*, *n* = 10, BL = 28.15 ± 3.9 cm and carp, *C. carpio*, *n* = 5, BL = 33.6 ± 2.1 cm) swimming in laminar flow conditions (control group, *n* = 15), and both in laminar and Kármán vortex flow conditions (treatment group, *n* = 6), as detailed in the electronic supplementary material, table S2. The Kármán vortices were generated using two different D-section cylinders: one with a diameter of 5 cm to produce relatively small vortices and another with a diameter of 7 cm for larger vortices. We conducted experiments at multiple flow speeds and analysed the data of 0.274 m s^−1^ because of the stronger effect of vortices. With our EMG platform, we collected 12-channel EMG signals synchronized with body kinematics data (see §4 for detailed experiment). We analysed the time lag or delay (∆*t*) based on EMG onset peaks and kinematics extrema (figure 1d; §4). A positive ∆*t* indicates that the EMG onset peak signal precedes the kinematics extrema, as shown in figure 4b, indicating the muscles are driving the body’s movement [21,23]. By contrast, a negative ∆*t* indicates that the kinematics precede the EMG, suggesting that the body moves first, with the muscles responding to proprioceptive sensing (figure 4e,h).

As a control, we first analysed the data with nine fishes collected for platform validation, where no vortices are involved. The flow was confirmed to be laminar with minimal boundary-induced distortion, as shown by two-dimensional Particle Image Velocimetry (PIV) measurements (see the electronic supplementary material, figure S17, video M4 and note S15). We initially focused on tail (caudal peduncle) movements, which are crucial for navigating complex flows (figure 4a) [3,39]. We observed that the fish EMG activity precedes body movement while having steady swimming in laminar flow, as evident from the probability density function of the F-distribution (f-PDF) of ∆*t* (tail lateral displacement (LD) versus L6/R6), as shown in figure 4c, where the majority of the ∆*t* are positive, with just 3.36% of ∆*t* being negative. The positive ∆*t* indicates that the EMG activity happened first, followed by the observable body movement. However, a shift happened in this typical pattern with the introduction of Kármán vortices in flow; the EMG activity would occasionally lag behind body movement, resulting in both positive and negative ∆*t* (electronic supplementary material, figure S16b,c). The f-PDF of ∆*t* (tail LD versus L6/R6), as shown in figure 4f, shows that there is a significant increase in the percentage of negative ∆*t*, with 48.38% of ∆*t* being negative when fishes are swimming in Kármán vortices, generated by a cylinder of diameter 5 cm. We then observed the ∆*t* in EMG activity and kinematics when fishes were swimming in Kármán vortices generated by a cylinder of diameter 7 cm while occasionally performing the Kármán gait. We found that the negative ∆*t* has decreased to 29.28%. We also observed similar trends, with a small occurrence of negative ∆*t* during laminar flow, while larger proportions of positive and negative ∆*t* were seen in Kármán vortices across all EMG channels, as shown in figure 4j–l. Notably, negative ∆*t* were more pronounced near the caudal peduncle (1 BL) and midbody region (0.5 BL), as detailed in the electronic supplementary material, table S8. This suggests that fish muscles may be activated, possibly passively, owing to body bending caused by the vortices, leading to a change in the pattern. Following this, the muscles may then actively drive body movement based on proprioceptive feedback.

We performed computational fluid dynamics (CFD) simulations based on experimentally recorded kinematics to further support these observations. These simulations indicated clear hydrodynamic interactions between the fish body and upstream-shed vortices (electronic supplementary material, figure S18, video M5). Notably, the lateral velocity ($U_{y}$) contours revealed a repeating pattern in which alternating regions of negative and positive lateral velocity closely matched the timing of the tail-beat cycle (electronic supplementary material, figure S19c,d). Negative $U_{y}$ regions corresponded to downward tail motion, while positive regions aligned with upward tail flaps. This phase alignment between vortex-induced flow and tail oscillation suggests that the fish tail may actively respond to or be influenced by these periodic hydrodynamic cues, further highlighting the role of proprioceptive feedback in responding to unsteady flows.

## 3. Discussion

This study provides experimental evidence for the proprioceptive sensing hypothesis in swimming fishes, by simultaneously recording synchronized multi-channel EMG and kinematics data and analysing the relationship between muscle activation and body movement under various flow conditions. We showed that in laminar flow, muscle activity consistently precedes body motion, whereas in vortex conditions, body bending can sometimes precede EMG activity, consistent with proprioceptive feedback. These findings suggest that fish muscles integrate both active propulsion and flow-induced sensing during locomotion. To achieve this, we developed and validated the custom-designed EMG–kinematics platform, which we confirmed using ML models that mapped EMG signals to joint angles and body pose with high accuracy. Beyond validating the platform, these analyses demonstrate its use for probing the role of proprioceptive feedback in shaping swimming behaviour under different flow regimes. Together, these results support the hypothesis that fish muscles function dually as actuators and proprioceptive sensors, enabling adaptation to complex flow environments.

Our platform demonstrates high-quality EMG-driven kinematic data collection, as evidenced by strong mapping of EMG to kinematics. We found that during steady swimming in laminar flow conditions, the EMG activity happens first, followed by observable body movement. This typical pattern in fishes for different gaits is reported in other studies [18,19,21,23,27,28] indicating that muscles drive the motion, underscoring the synchronization between predicted and measured movements. The multi-channel EMG activity revealed that muscle activity begins in the anterior part of the body and progresses towards the posterior, ending almost simultaneously on one side during steady swimming, similar to observations in other fish species ([23,27]; electronic supplementary material, figure S15). This pattern underscores the necessity for a sufficient number of channels to accurately capture body kinematics, which is evidenced by the improved mapping performance and low RMSE for joint angles and body pose when using data from 12 channels. Similar approaches have been applied to map EMG signals to joint angles in human limbs, particularly for upper limb and lower limb movements [36–38], supporting the effectiveness of EMG-based mapping for kinematic prediction. In these studies, researchers typically used four to six EMG channels per joint angle prediction, achieving accurate mapping for joint movements. Our approach aligns with these findings, using three channels per joint angle and 12 channels to predict a total of four joint angles, which reinforces the validity of our methodology and underscores the platform’s robustness for accurate proprioceptive sensing analysis in fishes.

Our findings indicate that, during interactions with vortices, fish body kinematics occasionally precede EMG muscle signals. This observation suggests that the fish’s body may initially be passively deformed by nearby vortices (similar to a dead fish performing Kármán gait when interacting with Kármán vortices [3]), followed by subsequent muscle activation detectable as EMG signals. This is consistent with previous studies in various species, including humans, cats, fishes and lobsters, where deviations from this typical pattern occur owing to perturbation, animals may also detect passive movement resulting from such stimuli [10,29,32,40]. The observation of increased negative Δ*t* near the caudal peduncle (1 BL) and midbody region (0.5 BL) aligns with studies indicating that interactions with Kármán vortices significantly influence these body regions [3,6,39]. Instances where muscle signals lead body kinematics imply that muscle activation may be driven by the muscle sensory input, allowing fish to modulate body kinematics in response to proprioceptive cues actively.

Our CFD simulations suggest that Kármán vortices shed from the upstream D-section cylinder probably persist downstream and interact with the fish body. Both pressure field contours and localized fluctuations in the lateral velocity component support this interaction ($U_{y}$) near the tail, indicating flow–body coupling. These findings reinforce the core premise of our study—that unsteady flow conditions can modulate fish body kinematics and potentially influence neuromuscular activation through proprioceptive feedback mechanisms. Future work combining direct flow measurements (e.g. PIV) with muscle activity recordings could clarify the dynamics of flow-mediated sensorimotor control in aquatic animals.

In the future, we can use this EMG-based approach to study fish body stiffness and proprioception sensing of fishes with impaired lateral lines in hydrodynamic wakes. Moreover, we can also use this method in natural or wildlife scenarios because most studies regarding fish behaviour, kinematics and hydrodynamics are conducted inside the laboratory using cameras in water treadmills and fish tanks. These methods have limitations, such as fishes behaving differently in laboratory conditions compared with natural habitats, and it is challenging to video record fishes continuously in nature. However, this method can be potentially used in wildlife scenarios to study fishes in their natural habitat. Very small EMG recording and transmission devices [41,42] can be used to record the fish EMG signals in nature. These small EMG recording devices could be upgraded to record multi-channel EMG signals from the fish’s body. Those EMG recordings can be retrieved from the recording devices, and then the retrieved EMG signal is processed in a computer at the laboratory. The kinematics of fishes and all the other parameters can be easily computed using the methods mentioned in the article. It will be an essential tool for studying real fishes in great detail and aspects of fishes that have never been explored.

## 4. Material and methods

### (a) Animal care

All experimental procedures and animal care were approved by the Institutional Animal Care and Use Committee of the Peking University. Laboratory animal professional technical examination certificate number: TY2018466.

Koi (*C. rubrofuscus*) and carp (*C. carpio*) fish were acquired from the fish market for this work (electronic supplementary material, table S2 and note S2). The fishes were kept in a well-maintained tank (2 × 4 × 0.3 m) with a capacity of 2400 l of fresh water. One-third of the water in the tank was replaced every week with fresh, non-chlorinated water. The automatic feeder fed the fishes three times a day. In the meantime, the fish tank water is filtered by 6000 l h^−1^. The water temperature was controlled and kept at 20 ± 2°C. All fishes were kept in the tank for two months after the experiment and then released into the lake with other fishes.

### (b) Electromyography and kinematic data acquisition

To acquire EMG data, the fish was first anesthetized using a 160 mg l^−1^ solution of Tricaine mesylate (MS222), and its BL and weight were measured. Electrodes were placed along the left and right sides of the fish’s body from 0.5 to 1.0 BL, based on the carangiform swimming mode of carp fish, which primarily uses the posterior musculature [43]. Electrode placements were equidistant along the midline, as outlined in the electronic supplementary material, table S1, with the reference electrodes placed above the gills (electronic supplementary material, figure S2).

Thin copper wire electrodes were hooked and inserted into the muscle through a hypodermic needle, allowing them to stay in place after withdrawal. Owing to challenges at high swimming speeds—such as electrode dislocation from drag and motion—we refined our electrode fixation method through trial and error. Electrodes were sutured to a dorsal fin ray and glued together for stability, minimizing motion artefacts and maintaining signal fidelity (see the electronic supplementary material, note S3 for details).

The set-up included two commercial biosignal amplifiers (ED0039), each acquiring six channels from the left and right sides of the fish (12 total), powered by an isolated DC source (electronic supplementary material, figures S2 and S3). Amplified signals were digitized using a National Instruments USB-6210 Data Acquisition (DAQ) device at a sampling rate of 2.5 kHz (16-bit resolution).

Following electrode placement, the fish recovered in a water treadmill (Loligo®) for 30 min before data collection began. EMG signals and kinematic recordings were obtained at five flow velocities (0.146, 0.184, 0.235, 0.265 and 0.274 m s^−1^). Kinematics were captured using a Sony RX100m5 camera mounted above the treadmill at 100 fps (figure 1a,b). Full hardware details and experiment protocols are provided in the electronic supplementary material, note S3.

### (c) Data processing

Raw EMG signals collected through biosignal amplifiers were digitized by the DAQ and processed using custom Python code for filtering, feature extraction and ML model development. A MATLAB [44] script was used to post-process fish kinematic data and compute parameters such as tailbeat frequency, displacement, amplitude and St from ML predictions.

EMG signals were first mean-adjusted, notch-filtered (50 Hz) and bandpass filtered (20–450 Hz) to remove noise and motion artefacts. High-quality recordings were confirmed by a mean signal to noise ratio (SNR) of 172.25 (linear), 19.18 dB (log) and low mean crosstalk of −0.158 between channels (SNR: *n* = 3; crosstalk: *n* = 4). EMG was downsampled from 2.5 to 1 kHz, while fish pose data were upsampled from 0.1 to 1 kHz to synchronize both datasets (see the electronic supplementary material, note S5)

A total of 18 EMG features were extracted from each channel (12 total), including time-domain, frequency-domain and time-frequency features (electronic supplementary material, table S3), resulting in a feature matrix $F_{j}$ for each channel j, as defined in the electronic supplementary material, equation S1. In parallel, the filtered EMG waveform was segmented using a 20-sample window to form signal matrices $S_{j}$ (electronic supplementary material, equation S2). These were concatenated into an augmented matrix $\left[F_{j}|S_{j}\right]$ per channel (electronic supplementary material, equation S3), and all channels were then stacked into the full EMG input matrix $V_{input}$ of shape (*n* × 456) (see the electronic supplementary material, notes S4 and S5, equation S4, figure S1g,h; figure 2a).

For kinematic data, DLC [35] was used to extract fish midline poses from high-speed videos (100 fps), based on 12 manually labelled points across the body (electronic supplementary material, figure S1c). DLC was trained using 1610 frames from 69 videos. Only pose data with a likelihood of greater than 0.9 was used and was interpolated using function Makima for stability. Pose data were transformed into joint space using a homogeneous transformation matrix (HTM) (electronic supplementary material, equation S5, figure S1d), and reduced to four joint angles representing the fish midline. These joint angles were resampled to match EMG timing and stacked into the output matrix $V_{output}$ (electronic supplementary material, equation S6, figure S1e,h; figure 2c).

### (d) Mapping electromyography to body kinematics

BPNNs, LSTMs and CNNs are used to predict the fish body. The experiment is conducted on nine fishes (subjects 1−9), as given in the electronic supplementary material, table S2, in laminar flow conditions at four water flow velocities for each fish in the water treadmill. The experiment was conducted more than once for some subjects and in some flow conditions, giving 44 datasets. The velocities in the water treadmill for fishes are 0.146,0.184, 0.235 and 0.274 m s^−1^. The datasets of eight subjects (40 datasets) are used to train the models, while the subject-3 dataset is used for testing the models.

The BPNN shown in the electronic supplementary material, figure S5 is an artificial neural network that uses a supervised learning algorithm to optimize the weights of its connections through the backpropagation of errors. The BPNN structure, as shown in the electronic supplementary material, figure S5, the code and the input size are shown in note S6. The BPNN model can use its powerful learning abilities to analyse muscle activity (EMG signals) and predict fish midline/body pose angles [36] by training on input variable shape (*n* × 456) (see the electronic supplementary material, figure S1h), paired with output variables, which are fish midline/body-pose ($\theta_{1},\theta_{2},\theta_{3},\theta_{4}$), where *n = 1,2,3,*… up to the length of the signal. BPNN can learn complex relationships between these input and output variables, enabling it to make accurate predictions and estimate the angles. This opens doors for exciting applications, although challenges like data quality and network design require careful consideration (see the electronic supplementary material, note S6)

The LSTM ML model, as shown in the electronic supplementary material, figure S6, is specialized for handling sequential (time series) data such as muscle activity [37]; for code, see the electronic supplementary material, note S7. LSTM excels at capturing temporal dependencies within EMG signals and can also be used to accurately predict fish body pose, where the order of muscle activations heavily influences movement. In a similar fashion to BPNN, LSTM is also trained; however, input variables shown in the electronic supplementary material, figure S1h are transformed into a format suitable for LSTM. The transformation involves specifying the number of time steps for each input sequence and creating a three-dimensional array where each slice along the first dimension corresponds to a sequence of consecutive rows from the original matrix. The resulting three-dimensional array of LSTM input is shaped (*n* × 20 × 456), where n, 20, and 456 correspond to batch size, time steps, and input variables, respectively (see the electronic supplementary material, note S7, figure S6).

CNN, as shown in the electronic supplementary material, figure S7 and code, as given in note S7, are designed to process and analyse visual data effectively [45]. CNNs are used to analyse input variables modelled as images (20 × 456 × 1). The network is trained on datasets containing spatial information and corresponding joint angles, enabling it to learn complex mappings between visual features in input variables and fish body pose movements. This approach is instrumental in applications like pose estimation, where understanding the fish body pose angles is essential. The CNN can contribute to predicting the fish body pose (angles) robustly and accurately. Like LSTM, a suitable input to CNN is formed by transforming a two-dimensional matrix representing input variables, as shown in the electronic supplementary material, figure S7, and then transforming it into a shape (batch size, time steps, features, 1). The resulting four-dimensional (4D) tensor has dimensions (*n* × 20 × 456 × 1), where each slice (20 × 456 × 1) represents a segment of the original input variables—iterating through all input variables and reshaping the input variables to populate a four-dimensional tensor (see the electronic supplementary material, note S8).

### (e) Computation of body pose and kinematics parameters

To reconstruct the fish’s body pose, we used a custom MATLAB [44] code that applies forward kinematics to the predicted joint angles. This process involves treating each body segment as a rigid link of length *L* and using the HTM defined in the electronic supplementary material, equation S5 to determine the position and orientation of each segment relative to the head-attached frame, as depicted in figure 3a. This approach enables the computation of the fish’s overall kinematics and other related parameters. The fish swimming for one tail-beat cycle of 0.612 s is shown in the electronic supplementary material, figure S13c, while the predictions of 20 s swimming along with real fishes are shown in the electronic supplementary material, video M2. The fish’s body bends laterally in a wave form, and the tip of the deflected curve is the tail, thus producing thrust to move forward, as illustrated in the electronic supplementary material, figure S13c. The body pose or midline points are interpolated with Makima function, as shown in the electronic supplementary material, figure S13c, to produce a smooth midline.

### (f) Experiments for the proprioceptive sensing hypothesis

Each fish was placed in a water treadmill filled with water from their home tank, ensuring environmental consistency. We measured water velocity at the centre of the test section using a turbine-type flow probe (LS300A) in the absence of the fish. Although these measurements were performed without the fish present, the average blockage ratio (BR) in our experiments was approximately 8% (electronic supplementary material, table S9), which is within the generally accepted threshold for minimizing flow distortion in swim tunnel studies [46,47]. Initially, fishes were allowed to acclimate by swimming in a laminar flow at 0.124 m s^−1^ for 30 min. After acclimation, the water treadmill flow rate was increased to a specified speed under laminar conditions, and the fish swam for 1 min before EMG and kinematic data were collected for an additional one or more minutes in each condition (figure 4a).

Following the laminar flow trials, the water flow rate was reduced to the initial 0.124 m s^−1^ and a D-section cylinder [22] with a diameter of 5 cm was introduced to generate Kármán vortices, as shown in figure 4d,g (see the electronic supplementary material, videos M3 and M6 for Kármán vortex generation). Fishes were allowed to swim in this vortex environment for 5 min before data collection resumed under specific turbulent flow conditions. Subsequently, the flow rate was increased to predetermined values while maintaining the vortex street, and EMG and kinematic data were collected for one or more minutes to capture muscle activation and body dynamics as fishes interacted with the vortex flow. This procedure was repeated with a 7 cm diameter D-section cylinder, providing a range of vortex conditions for proprioceptive analysis. By comparing EMG and kinematic data across these distinct flow conditions, we obtained a comprehensive dataset on muscle and movement patterns in response to laminar and turbulent flows (electronic supplementary material, table S2).

To evaluate the influence of upstream-shed Kármán vortices on the fish body and the potential for flow-induced modulation of muscle activity, we performed CFD simulations using the IBAMR framework (electronic supplementary material, note S16). These simulations incorporated experimentally recorded kinematics of freely swimming fishes and enabled direct comparisons of flow fields under different conditions, including with and without wall effects and with or without an upstream D-section cylinder. The first goal of CFD was to quantify the blocking and wall effects, and secondly, to determine whether vortex–body interactions occurred and to assess their potential role in modulating proprioceptive feedback.

We assessed potential wall-induced effects in the flow tank. We first computed the dynamic BR using the projected area of the fish body. The mean BR was approximately 8%, slightly above the 5% engineering ideal but within the commonly accepted 10% threshold in fish swimming studies [46,47], where wall effects are considered minimal. To further evaluate confinement effects, we performed CFD simulations of the fish swimming with identical kinematics under two boundary conditions: one with no-slip walls replicating the physical tank, and another with slip (symmetry) boundaries to eliminate wall influence (electronic supplementary material, note S16, figure S18a,b and video M5). In the absence of walls, the upstream Kármán vortices showed greater lateral dispersion and appeared more diffused. With walls present, the vortices remained more confined near the centreline and retained stronger coherence. Despite these differences, the vortices consistently propagated downstream and interacted with the fish body in both cases (electronic supplementary material, figure S18c), indicating that the essential flow–body interactions observed experimentally were preserved.

The CFD results confirmed robust hydrodynamic interactions between the shed vortices and the fish body. Pressure field contours (electronic supplementary material, figure S18 and video M5) revealed that the Kármán vortices generated by the cylinder propagated downstream and circulated along the body of the swimming fish (electronic supplementary material, figure S19c), suggesting persistent flow–body coupling. Additionally, analysis of the lateral velocity component ($U_{y}$) (electronic supplementary material, figure S19 and video M6) showed that in the presence of the cylinder, there were noticeably stronger lateral flow fluctuations near the fish’s body. These fluctuations were absent or markedly weaker in the cylinder-free case. However, we observed a repeating pattern where alternating lateral flow (blue to red regions) coincided with the fish’s tail-beat cycle (electronic supplementary material, figure S19c,d). Specifically, negative lateral velocity (blue) aligns with the tail being pushed downwards, followed by a positive velocity region (red) as the tail flaps upwards. This alternating flow pattern appears to match the phase of the tail’s oscillation, suggesting that the tail may be responding to, or synchronizing with, the periodic vortices shed by the upstream cylinder. Such asymmetric or unsteady flow inputs near the tail region could plausibly alter body kinematics and trigger proprioceptive reflexes, which in turn could influence subsequent EMG activity.

To characterize vortex generation, we conducted dye visualization experiments confirming the generation of Kármán vortices from the D-section cylinder without the fish present (electronic supplementary material, video M3). Owing to ethical and practical limitations, such as potential fish stress, behavioural interference and rapid dye dispersion under unsteady conditions, we did not apply dye to the fish in the flow. Instead, as a preliminary exploration, CFD simulations incorporating realistic swimming kinematics were used to visualize vortex propagation and interaction with the body. While these simulations support our hypothesis of flow–body interaction, we acknowledge the absence of direct flow measurements around the fish as a limitation.

### (g) Calculation of delay ∆t

To analyse the temporal relationship between muscle activation and body LD, raw EMG signals were first processed through a standardized signal conditioning pipeline (figure 1d). The signal was detrended to remove linear drift, followed by a bandpass filter (to eliminate motion artefacts and suppress high-frequency noise) and a notch filter to eliminate power line interference. The resulting signal was then rectified and smoothed using a moving average to generate the EMG envelope representing muscle activity (figure 1d(i–iv)):


$$\Delta t_{k}^{left}=\;\left\{ \begin{array}{c} \Delta t_{k}^{LD,\;min}-t_{before}^{EMG,L},\;if\;\left|\Delta t_{k}^{LD,\;min}-t_{before}^{EMG,L}\right|-\;\left|\Delta t_{k}^{LD,\;min}-t_{after}^{EMG,L}\right|<\epsilon \\ \Delta t_{k}^{LD,\;min}-t_{after}^{EMG,L},\;\text{otherwise} \end{array}\right.,$$

$$\Delta t_{k}^{right}=\;\left\{ \begin{array}{c} \Delta t_{k}^{LD,\;max}-t_{before}^{EMG,R},\;if\;\left|\Delta t_{k}^{LD,\;max}-t_{before}^{EMG,R}\right|-\;\left|\Delta t_{k}^{LD,max}-t_{after}^{EMG,R}\right|<\epsilon \\ \Delta t_{k}^{LD,\;max}-t_{after}^{EMG,R},\;\text{otherwise} \end{array}\right..$$

EMG onset peaks were detected using a prominence and separation-based local maxima search (figure 1d(v)). Simultaneously, maxima and minima of the LD were identified from kinematic data (figure 1d(vi)). For each LD minima, the nearest left EMG onset peak or LD maxima, the nearest right EMG onset peak was located either before or after the kinematic event (figure 1d). The delay was computed as the time difference between the LD maxima/minima and the nearest left/right EMG onset peak. A small threshold (*ε* = 10 ms) was used to resolve ambiguous cases, selecting the temporally closer EMG event. This allowed us to estimate the delays ($∆t_{k}^{left}$, $∆t_{k}^{right}$) between muscle activation and resulting body LD motion on both the left and right sides (see the electronic supplementary material, note S14 for details).

## Data Availability

The code file and datasets are published at Zenodo [48]. Supplementary material is available online [49].
